# MAP-ping genomic organization and organ-specific expression profiles of poplar MAP kinases and MAP kinase kinases

**DOI:** 10.1186/1471-2164-7-223

**Published:** 2006-08-31

**Authors:** Marie-Claude Nicole, Louis-Philippe Hamel, Marie-Josée Morency, Nathalie Beaudoin, Brian E Ellis, Armand Séguin

**Affiliations:** 1 Natural Resources Canada, Canadian Forest Service, Laurentian Forestry Centre, 1055 du P.E.P.S., P.O. Box 10380, Stn. Sainte-Foy, Quebec, Quebec, G1V 4C7, Canada; 2 Michael Smith Laboratories, University of British Columbia, 2185 East Mall, Vancouver, British Columbia, V6T 1Z4, Canada; 3 Département de biologie, Université de Sherbrooke, Sherbrooke, Quebec, J1K 2R1, Canada

## Abstract

**Background:**

As in other eukaryotes, plant mitogen-activated protein kinase (MAPK) cascades are composed of three classes of hierarchically organized protein kinases, namely MAPKKKs, MAPKKs, and MAPKs. These modules rapidly amplify and transduce extracellular signals into various appropriate intracellular responses. While extensive work has been conducted on the post-translational regulation of specific MAPKKs and MAPKs in various plant species, there has been no systematic investigation of the genomic organization and transcriptional regulation of these genes.

**Results:**

Ten putative poplar *MAPKK *genes (*PtMKKs*) and 21 putative poplar *MAPK *genes (*PtMPKs*) have been identified and located within the poplar (*Populus trichocarpa*) genome. Analysis of exon-intron junctions and of intron phase inside the predicted coding region of each candidate gene has revealed high levels of conservation within and between phylogenetic groups. Expression profiles of all members of these two gene families were also analyzed in 17 different poplar organs, using gene-specific primers directed at the 3'-untranslated region of each candidate gene and real-time quantitative PCR. Most *PtMKKs *and *PtMPKs *were differentially expressed across this developmental series.

**Conclusion:**

This analysis provides a complete survey of *MAPKK *and *MAPK *gene expression profiles in poplar, a large woody perennial plant, and thus complements the extensive expression profiling data available for the herbaceous annual *Arabidopsis thaliana*. The poplar genome is marked by extensive segmental and chromosomal duplications, and within both kinase families, some recently duplicated paralogous gene pairs often display markedly different patterns of expression, consistent with the rapid evolution of specialized protein functions in this highly adaptive species.

## Background

Members of the mitogen-activated protein kinase family are involved in major signaling pathways in all eukaryotes [1]. These pathways, which are typically activated by intracellular or environmental cues, usually consist of three hierarchically organized protein kinases. The first component of this module, the MAPK kinase kinase (MAPKKK), activates a downstream MAPK kinase (MAPKK) through double serine-threonine phosphorylation. The phosphorylated MAPKK then acts as a dual-specificity protein kinase to activate the third component of the pathway, *i.e*. MAPK, via phosphorylation of specific threonine and tyrosine residues in a T-X-Y motif located within the activation loop of the protein. At this point, activated MAPKs can modulate various cellular activities through activation of other protein kinases, or metabolic enzymes, or by phosphorylation of transcription factors and components of the cytoskeleton. Important links between MAPK activities and fundamental processes like cell proliferation/differentiation and defence responses have been established from extensive studies performed in human, mouse and yeast systems [2].

MAPK cascades are also present in plants [3-5], where they have been involved in a wide variety of phenomena, including plant responses to biotic, abiotic and oxidative challenges [6-9], hormone signaling [10-12], plant cytokinesis [13] and pollen development [14]. The *Arabidopsis thaliana *genome encodes at least 60 MAPKKKs, 10 MAPKKs and 20 MAPKs [4] but most of these proteins have not been functionally characterized. The plant MAPKK and MAPK families have both diverged into four major groups (A, B, C and D). MAPKs belonging to groups A, B and C all possess a TEY motif in their activation loop, while members of group D harbor a TDY motif. The most extensively studied plant MAPKs are *Arabidopsis *AtMPK3 and AtMPK6, and their *Nicotiana tabacum *orthologs, NtWIPK and NtSIPK, respectively. These group A MAPKs have been involved in non-host disease resistance [15,16], gene-for-gene defence signal transduction [17,18], wounding response [19] and ethylene production [20,21]. They also appear to act as positive regulators of the hypersensitive response (HR) [22], a defence-related form of programmed cell death. In rice (*Oryza sativa*), several MAPKs have also been characterized and display similar stress response functions, as well as developmental regulation [23-25].

The cellular functions in which MAPKs participate are mainly dependent on their phosphorylation status. In single-celled organisms like yeast, post-translational mechanisms seem to account for most of the regulation of the MAPK cascades, with little evidence of transcriptional regulation. On the other hand, in multicellular eukaryotes, MAPK cascades regulation often occurs at the transcriptional, post-transcriptional and post-translational levels [26]. In mammals, the duration of MAPK activation can depend on the nature of the stimulus, and these differences in the temporal pattern of activation can lead to distinct physiological responses in the cell [27]. This has also been demonstrated in tobacco, where transient activation of NtMEK2 (a stress-responsive MAPKK) and its downstream effector, NtSIPK, induces strong expression of defence-related genes, whereas sustained activation of the same proteins leads to the activation of NtWIPK and subsequent cell death [22]. Compartmentalization and organization of yeast and mammalian MAPK cascade components by scaffolding proteins [28,29]. can also contribute to signaling specificity [2]. For plants, there is no published data involving scaffolding proteins in MAPK signaling, but a recent report has described the formation of protein complexes that include stress responsive MAPKs [30]. Subcellular compartmentalization of MAPK components may also be critical to their function in plants, since treatment of *Petroselinum crispum *cells with a *Phytophthora*-derived elicitor resulted in the translocation of three cytosolic MAPKs to the nucleus, where they are thought to interact with transcription factors [31].

Regulation of MAPKs at the transcriptional and post-transcriptional levels can also play an important role in controlling the MAPK cascades function. Alternative splicing has been observed for the mammalian MAPK ERK1 [26,32], as well as for the *Arabidopsis MAPKKK *gene, *ANP1 *[33], and the rice *MAPK *gene, *OsMPK5 *[34]. Moreover, strong up-regulation in the expression of some plant *MAPK *genes is seen in response to stress, including tobacco *NtWIPK *[15], tomato *LeMPK3 *[8], alfalfa *MsMMK4 *[35] and rice *OsBWMK1 *[36] and *OsMSRMK3 *[25]. Finally, some plant *MAPKs *display organ-specific expression, suggesting that their function is spatially and/or temporally delimited. For example, the *Petunia hybrida MAPK *gene, *PMEK 1*, is preferentially expressed in reproductive female organs [37], while the tobacco *MAPK *gene *Ntf4 *(a close relative of the stress-responsive *MAPK *gene *NtSIPK*) is expressed only in certain organs such as pollen grains, developing embryos and mature embryos [38].

Transcriptional regulation of MAPK cascade components thus appears to provide an important level of control in plants, suggesting that systematic analysis of their transcriptional patterning in a given plant species should provide insight into potential biological functions of specific classes of these signaling components. The development of transcriptomic databases (microarray and others) in some model plant species such as *Arabidopsis *and rice has made it possible to track, *in silico*, the expression profile of a given *MAPK *gene. The recent availability of a genome sequence from *Populus trichocarpa *now opens up the possibility to investigate, based on transcriptional regulation, the possible involvement of specific *MAPK *gene family member(s) in organ development processes that are unique to woody species. In this paper, we conducted a comprehensive analysis of the organ-specific expression patterns for all predicted poplar *MAPK *and *MAPKK *genes. Correlation of these data with structural analysis of both the *MAPKK *and the *MAPK *gene families also revealed distinct expression patterns within recently duplicated (paralogous) gene pairs, suggestive of rapid evolution of specialized signaling protein functions in this highly adaptable woody perennial.

## Results

### Genomic distribution of poplar MAPK and MAPKK genes

**Figure 1:**
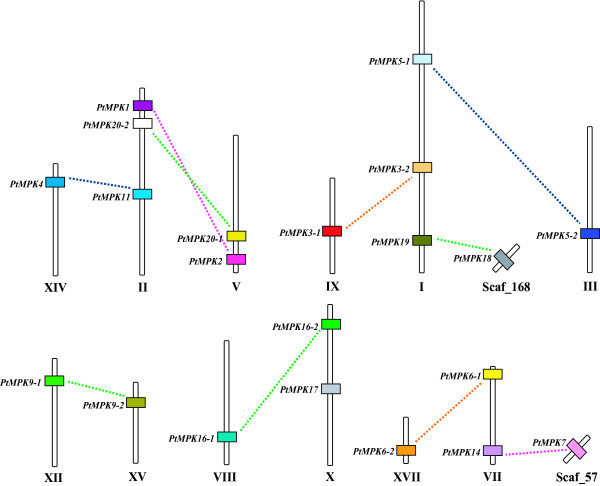
Schematic view of the scattered distribution of the poplar *MAPK *genes (*PtMPKs*) over the *Populus trichocarpa *genome. Twelve of the 19 poplar chromosomes are presented as vertical bars. In addition, two scaffolds containing a *PtMPK *gene are shown. *PtMPK *genes are represented by colored boxes and the color code presented here is also used in the other figures. Recent duplication events between paralogous genes are indicated using dotted lines.

Previous analysis of the genome sequence of poplar (*Populus trichocarpa*) had identified robust gene models corresponding to all the *MAPK *(*PtMPK*) and *MAPKK *(*PtMKK*) family members [39]. With this information, we were able to obtain an overview of the chromosomal distribution of these important signaling components. *PtMPK *genes are distributed over 12 of the 19 poplar chromosomes (Figure 1). Chromosomes I and II both carry three divergent *PtMPK *genes, whereas chromosomes V, VII and X display two *PtMPK *genes each. The remaining poplar *MAPK *genes are unique with respect to their chromosomal location. *PtMPK7 *and *PtMPK18 *have not yet been assigned to any linkage group, and therefore remain positioned on their respective scaffolds. Interestingly, although there are numerous *PtMPK *paralogs displaying high levels of sequence similarity, they are distributed all across the genome and do not form clusters containing closely related genes as may have been expected if they originated from local duplication events. This pattern probably reflects the series of whole genome, chromosomal and large segmental duplication events that typify the poplar genome (G. Tuskan, personal communication).

**Figure 2:**
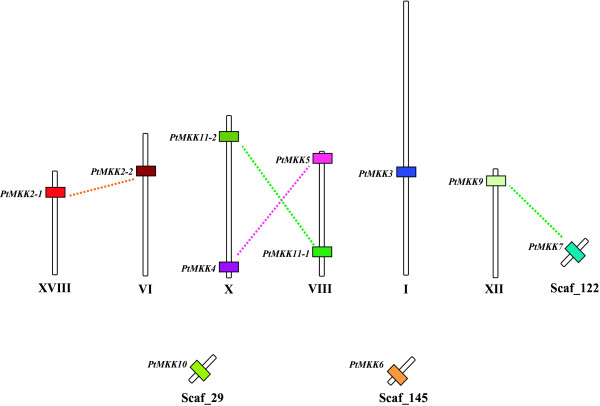
Schematic view of the scattered distribution of the poplar *MAPKK *genes (*PtMKKs*) over the *Populus trichocarpa *genome. Six of the 19 poplar chromosomes are presented as vertical bars. In addition, three scaffolds containing a *PtMKK *gene are shown. *PtMKK *genes are represented by colored boxes and the color code presented here is also used in the other figures. Recent duplication events between paralogous genes are indicated using dotted lines.

*PtMKK *genes also display a scattered genomic distribution (Figure 2) across six of the 19 poplar chromosomes, with three (*PtMKK6*, *PtMKK7 *and *PtMKK10*) of the 11 gene models located on unattributed scaffolds. Only chromosomes VIII and X contain more than one *PtMKK *gene.

### Exon and intron organization of poplar MAPK and MAPKK genes

**Figure 3:**
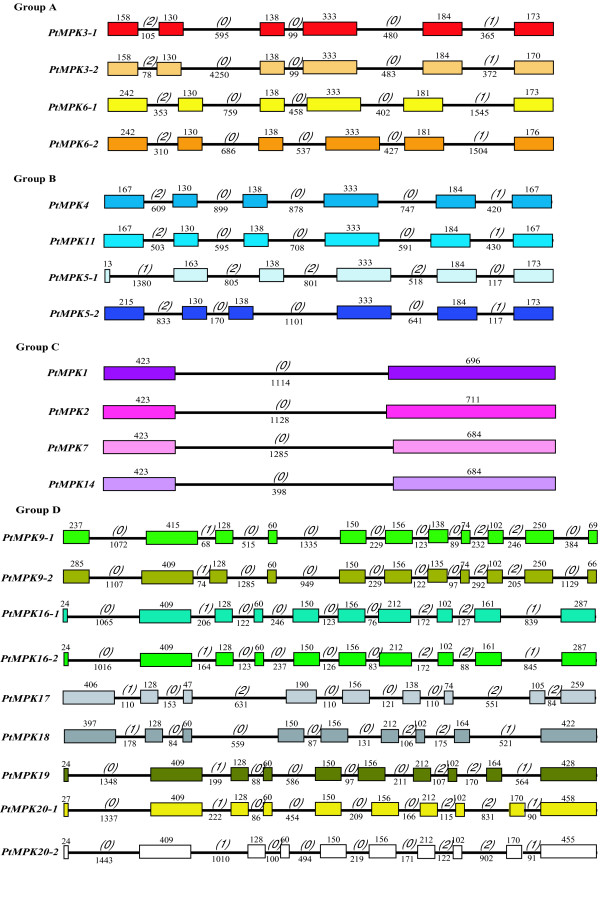
Intron and exon organization of poplar *MAPK *genes (*PtMPKs*). Introns and exons are represented by black lines and colored boxes respectively. The length in base pairs of each intron and exon is also indicated. Numbers between brackets correspond to the intron phase. *PtMPKs *have been grouped according to phylogenetic classification [39].

**Figure 4:**
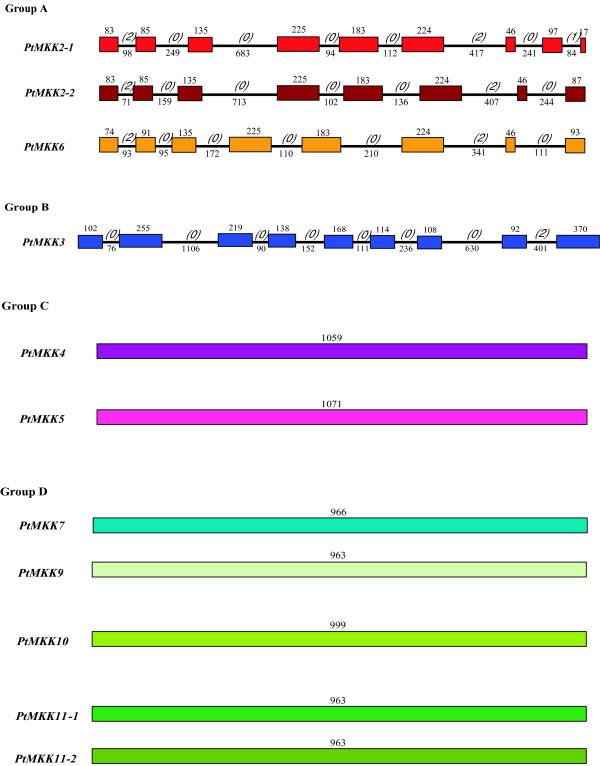
Intron and exon organization of poplar *MAPKK *genes (*PtMKKs*). Introns and exons are represented by black lines and colored boxes respectively. The length in base pairs of each intron and exon is also indicated. Numbers between brackets correspond to the intron phase. *PtMKKs *have been grouped according to phylogenetic classification [39].

**Figure 5:**
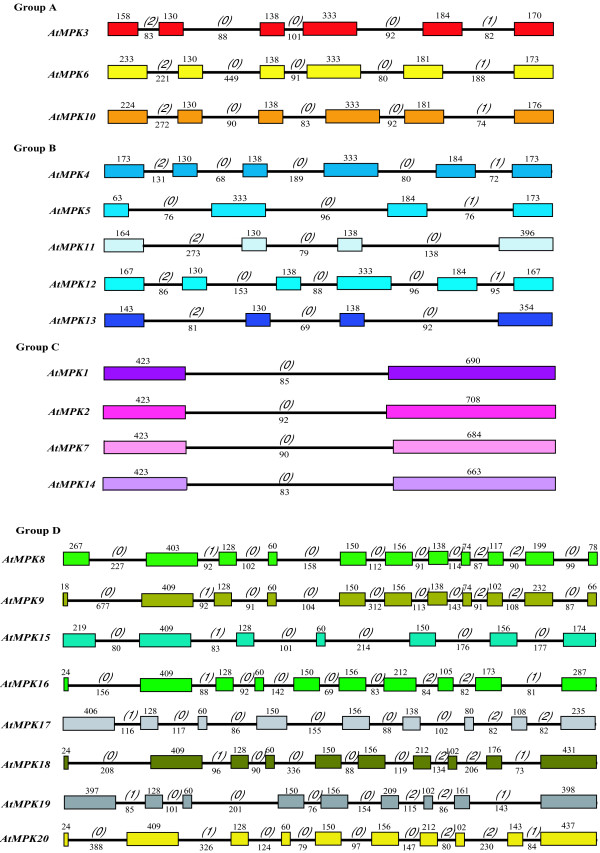
Intron and exon organization of *Arabidopsis MAPK *genes (*AtMPKs*). Introns and exons are represented by black lines and colored boxes respectively. The length in base pairs of each intron and exon is also indicated. Numbers between brackets correspond to the intron phase. *AtMPKs *have been grouped according to phylogenetic classification [39].

**Figure 6:**
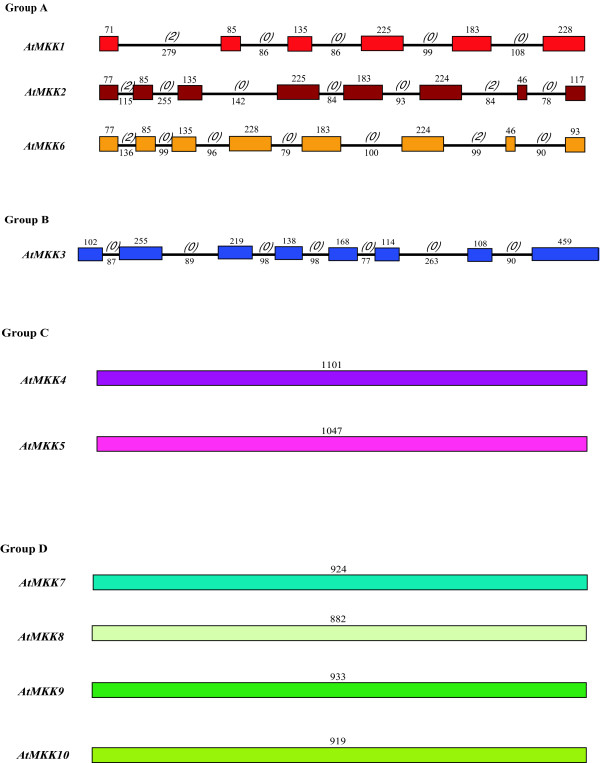
Intron and exon organization of *Arabidopsis MAPKK *genes (*AtMKKs*). Introns and exons are represented by black lines and colored boxes respectively. The length in base pairs of each intron and exon is also indicated. Numbers between brackets correspond to the intron phase. *AtMKKs *have been grouped according to phylogenetic classification [39].

Analysis of the pattern of exon-intron junctions can provide important insights into the evolution of gene families. Therefore, we extracted data regarding predicted exon and intron distribution for the coding regions of all *PtMPKs *and *PtMKKs *(Figures 3 and 4) as well as for all *Arabidopsis *putative orthologs (*AtMPKs*, Figure 5 and *AtMKKs*, Figure 6). Group A *PtMPKs *exhibit a highly conserved distribution of exons and introns (Figure 3) consisting of six exons of conserved length, and five introns of conserved or variable sizes. *PtMPKs *belonging to group B also possess six exons, with lengths similar to those found in group A *PtMPKs*, while the associated introns vary in size between the different members of group B. Group C *PtMPKs *are each composed of only two exons with strictly conserved or very similar sizes. *PtMPK14 *is the only group C member with a shorter intron (398 *vs *~ 1200 base pairs for the other three members).

In contrast to these three highly conserved structural patterns, group D *PtMPKs *possess a complex distribution of exons and introns, including different pattern subsets within the same phylogenetic group. For instance, *PtMPK9-1 *and *PtMPK9-2 *are both composed of 11 exons, whereas *PtMPK16-1*, *PtMPK16-2*, *PtMPK19*, *PtMPK20-1 *and *PtMPK20-2 *all possess ten. Despite some modest differences in the length of particular exons, it is clear that the exon structural pattern is well conserved not only between close paralogs (e.g. *PtMPK16-1 *and *PtMPK16-2*), but also between group D *PtMPKs *that apparently diverged following earlier duplication events (e.g., *PtMPK16-1 *and *PtMPK19*). These same patterns are also found in the *Arabidopsis MPK *gene family with the exception of group B *MPKs*, which display three different patterns of exon-intron distribution (Figure 5).

The *MKK *genes display two strikingly different structural patterns in both poplar and *Arabidopsis *(Figures 4 and 6). Members of group C and D *MKKs *have a completely intronless configuration, whereas the group B *MKK3s *and all group A *MKKs *possess numerous exon and intron junctions. In poplar, *PtMKK2-1*, *PtMKK2-2 *and *PtMKK6 *show quite strong exon length conservation, with the exception of an additional 17 base pairs exon in *PtMKK2-1*. *PtMKK3*, on the other hand, has a completely unique exonic structure, consistent with both its evolutionary distinctiveness [39] and the presence of an unusual C-terminal NTF2 domain that is not found in any other MKK group.

Intron phase (i.e., the position of an intron within a codon; phase 0 when lying before the first base, phase 1 when lying after the first base and phase 2 when lying after the second base) was also assessed for all *PtMPK *and *PtMKK *gene models (Figures 3 and 4), as well as for all *AtMPK *and *AtMKK *gene models (Figures 5 and 6). For both *PtMPKs *(58%) and *PtMKKs *(73%), the majority of introns are within phase 0, while 23% of introns found in both poplar protein kinase families are within phase 2. Phase 1 introns represent 19% of all *PtMPKs *introns and only 4% of all *PtMKKs *introns. For *Arabidopsis*, similar numbers are found (Figures 5 and 6), except that there are no phase 1 introns predicted within *AtMKK *genes.

The association of two adjacent introns in eukaryotic genes can be in any of nine different intron phase combinations, leading to two classes of exons: symmetric exons (0-0), (1-1), (2-2) and asymmetric exons (0-1), (0-2), (1-0), (1-2), (2-0), (2-1). For poplar *MPKs*, 51% of all exons are symmetric and the great majority of these are (0-0) exons (79%). A similar picture is found in *Arabidopsis*, where 54% of *AtMPKs *exons are symmetric. Once again the majority of these are (0-0) exons (85%). No symmetric exons harboring the (1-1) configuration are found in any of the poplar or *Arabidopsis MPK *gene models. For the *PtMKK *and *AtMKK *gene families, we respectively found 58% and 68% of symmetric exons and for both species, all of these symmetric exons are in the (0-0) configuration. For both plants, the most frequent asymmetric exons found in *MPKs *and *MKKs *are those belonging to the (0-1), (0-2) and (2-0) configurations.

### Real-time quantitative PCR data normalization and general considerations

The real-time, fluorescence-based reverse transcription polymerase chain reaction (RTqPCR) technique has become a method of choice because of its wide dynamic range, sensitivity and robust quantification of mRNA levels. In contrast to microarray profiling, transcript accumulation (TA) of closely-related gene members can also be easily discriminated in RTqPCR by using oligonucleotide primers specific to unique gene signatures. Such comparisons are only valid, however, if the chosen primer sets display comparable efficiencies in their ability to amplify targeted amplicons. We therefore tested all the selected primer sets against genomic DNA from three different genetic backgrounds, namely *Populus trichocarpa *(Nisqually-1), *Populus trichocarpa X Populus deltoides *(H11-11) and *Populus deltoides *(ST-70). In these assays, most primer sets yielded a very similar Ct value (around 21 for most genes) across the three different genetic backgrounds (see additional file 1). This indicates that these primer pairs can equally hybridize to the *P. trichocarpa *alleles in the Nisqually-1 background, to the *P. deltoides *alleles in the ST-70 background and most importantly to both the *P. trichocarpa *and the *P. deltoides *alleles in the H11-11 hybrid, which was used in this study to monitor *PtMPK *and *PtMKK *expression profiles. On the other hand, primer pairs specific for *PtMPK2*, *PtMPK4*, *PtMPK9-2*, *PtMPK16-2*, *PtMKK4 *and *PtMKK7 *all display higher Ct values in the *P. deltoides *background. This reflects that these gene specific primer pairs preferentially hybridize to the *P. trichocarpa *allele in the hybrid genotype, and that TA obtained on cDNA (see below) might slightly underestimate the actual level of expression since the *P. deltoides *allelic contribution is not perfectly captured. This observation could be related to lower primer set efficiency, resulting from polymorphism within the 3'UTR region of the different alleles. Nevertheless, melting curve and gel electrophoresis analysis confirmed single product amplification for all respective *MPKs *and *MKKs *in the three poplar genotypes (data not shown). Moreover, within one particular genetic background, the steady Ct value obtained for the different *MPKs *and *MKKs *confirmed that for a constant number of target sequences (e.g., 5 ng of DNA), each primer set gave a similar Ct value (see additional file 1). This clearly demonstrates the similar efficiencies of the various primer sets used in this study, and allows direct gene-to-gene comparisons of the levels of expression detected in the various organs sampled.

**Figure 7:**
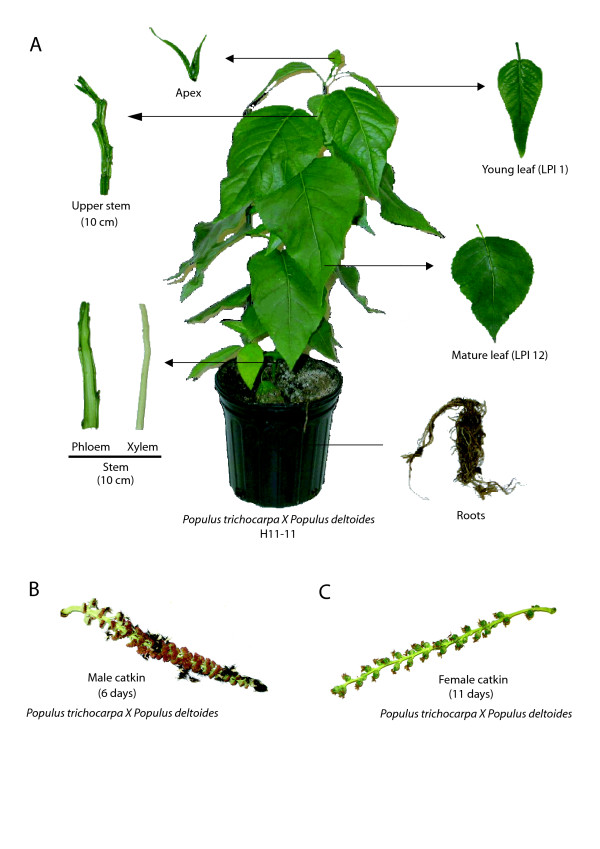
Illustration of some of the harvested poplar organs used in this study. **(A) ***P. trichocarpa *X *P. deltoides*; H11-11 hybrid clone at PI 16 stage.**(B) **Harvested male catkin from cut branches of 10-year-old field-grown trees (*P. trichocarpa *X *P. deltoides*). **(C) **Harvested female catkin from cut branches of 10-year-old field-grown trees (*P. trichocarpa *X *P. deltoides*).

**Figure 8:**
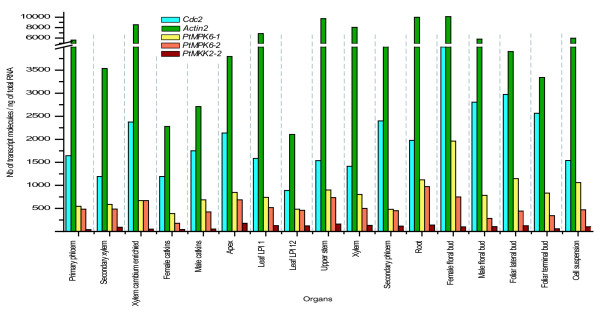
Steady-state transcript accumulation for *cdc2*, *Act2 *and three kinase genes throughout the surveyed organs (indicated at the bottom of the figure). After determination of RTqPCR primers efficiency and generation of standard curves, RTqPCR analysis was performed for *cdc2*, *Act2*, and for two poplar *MPKs *as well as for one poplar *MKK*. Twelve nanograms of cDNA were used in each RTqPCR reaction. Results are expressed in number of specific transcripts per ng of total RNA. Values represent the mean of six independent reactions (two repeats for each of three independent samples). Cts were determined using single fluorescent readings that were taken after each cycle.

Finally, the use of internal standard candidate genes that display relatively stable expression over time and between tissues or organs allows sample-to-sample normalization of the RTqPCR expression data. In the present study, the use of RTqPCR primers specific for a *cyclin-dependent kinase 2 *(*cdc2*) gene revealed generally consistent levels of *cdc2 *TA in different organs from poplar (Figure 7A, B, C), with less than two-fold variation observed across most vegetative organs, and in suspension-cultured cells (Figure 8). Slightly higher transcript abundance of *cdc2 *was detected in actively growing organs, such as floral and foliar buds, where CDC2 is likely involved in cell-cycle progression [40,41]. Therefore, *cdc2 *expression levels provided a good normalization baseline. In addition, since *Actin 2 *(*Act2*) has been stated to be a good internal control gene for RTqPCR studies across various poplar organs [42], we have used this candidate as housekeeping gene. TA for *Act2 *proved to be quite stable across most organs, with a maximum five-fold increase from mature leaf to upper stem (Figure 8). This level of variation seems relatively low considering the wide diversity of tissues tested in our survey (from meristematic organs such as buds to mature leaves). Moreover, our results revealed that two poplar *MAPKs *(*PtMPK6-1/6-2*) display quite constant TA in all assayed organs (Figure 8). The highest TA was detected in the female floral bud sample, but this level of expression represents only a one-fold increase in comparison to the average TA in all organs. Finally, *PtMKK2-2 *was also detected at low but constant levels in all tested organs. The respective TA levels for *cdc2*, *Act2*, *PtMPK6-1*, *PtMPK6-2 *and *PtMKK2-2 *across many different organ types provide confirmation that an appropriate and consistent dosage of cDNA was used in the various RTqPCR reactions. In our analysis, TA levels corresponding to <100 transcript molecules per ng total RNA were scored as 'very low', values of 100–400 as 'moderate', values >400 as 'high', and values >1000 as 'very high'. Levels <10 were treated as effectively zero.

### Poplar MAPK and MAPKK gene expression patterns

Virtually all *PtMPK*s and *PtMKK*s are expressed in all organs analyzed, but their level of expression varies considerably. Regardless of the phylogenetic groups or organs examined, the TA for *PtMPK*s generally fluctuates between moderate to very high levels (See additional file 2). Members of group D *MPKs *show TA levels of ~1500 transcript molecules per ng of total RNA, while members of the group A, B and C *MPKs *show lower levels, around 400–600. For the Pt*MKK *gene family, the most highly expressed members are those belonging to group C (TA >1600, see additional file 3). Group B and D *MKK *genes have similar levels of TA (~1100–1600). Finally, group A *MKK *genes are the most weakly expressed, with on average 550 transcript molecules per ng of total RNA.

### MAPKs

#### Group A MPKs

**Figure 9:**
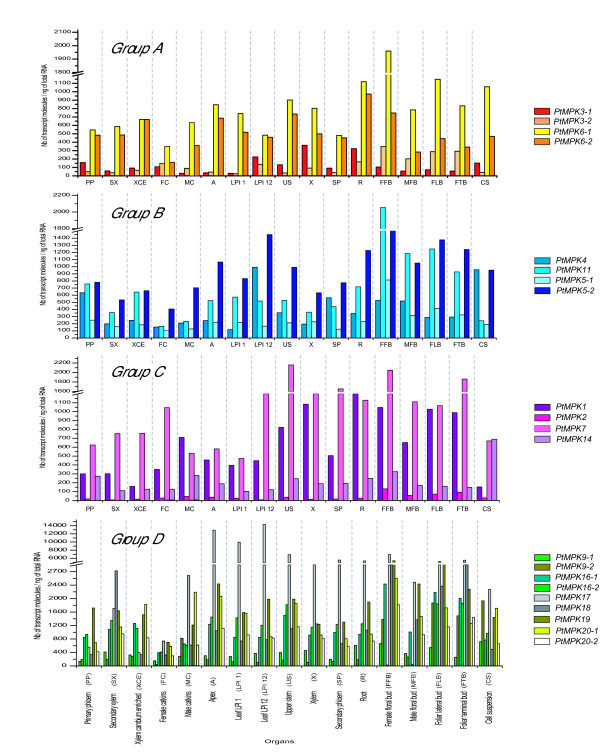
Steady-state transcript accumulation for all members of the four phylogenetic groups of *PtMPK *genes. After determination of RTqPCR primers efficiency and generation of standard curves, RTqPCR analysis was performed for each poplar *MAPK *gene. Twelve nanograms of cDNA were used in each RTqPCR reaction. Results are expressed in number of specific transcripts per ng of total RNA. Values represent the mean of six independent reactions (two repeats for each of three independent samples). Cts were determined using single fluorescent readings that were taken after each cycle.

The most extensively studied plant MAPKs belong to Group A which, in *Arabidopsis*, consists of three members, *AtMPK3*, *AtMPK6 *and *AtMPK10 *[4]. No direct putative ortholog of *AtMPK10 *has been detected in the poplar genome, but phylogenetic analysis [39] revealed the presence of two closely-related poplar presumed orthologs for the defense-related genes *AtMPK3 *(*PtMPK3-1 *and *3-2*) and *AtMPK6 *(*PtMPK6-1 *and *6-2*). In most organs, the expression of both *PtMPK3-1 *and *3-2 *is lower than that of *PtMPK6-1 *and *6-2 *(Figure 9; see additional file 2). *PtMPK3-1 *is relatively strongly expressed in roots and xylem, in comparison to other samples. In most organs *PtMPK3-1 *tends to be slightly more expressed than *PtMPK3-2*. However, this pattern is reversed in the four types of buds (male and female floral buds, lateral and terminal foliar buds) as well as in both types of catkins. *PtMPK6-1 *and *6-2 *both show similar expression profiles across many organs, but TA for *PtMPK6-1 *becomes slightly more pronounced than that of its paralog in all four types of buds, in both types of catkins and in suspension-cultured cells.

#### Group B MPKs

There is less information on the biological roles of the other MAPK groups in plants, although some reports have suggested the potential involvement of group B *MPK *genes in response to environmental stresses as well as in cell development [4]. In poplar, *PtMPK11 *and *PtMPK5-2*, the most highly expressed of the four group B *MAPK *genes, are particularly actively transcribed in male and female floral buds (Figure 9). By contrast, the paralogous *PtMPK5-1 *gene shows the lowest level of TA within this group.

#### Group C MPKs

Among the group C *MPKs*, it has been reported that the tobacco *Ntf3 *gene is expressed in pollen [43] and that the *Arabidopsis AtMPK7 *gene has circadian rhythm-regulated patterns of expression [44]. The most highly expressed of the four poplar group C *MPK *genes is *PtMPK7*, with elevated TA levels detected in female catkin, buds, phloem, xylem, mature leaves (LPI 12) and roots (Figure 9). This gene is also differentially expressed in particular developmental stages of specific organs, with more abundant transcripts detected in floral buds (male and female) than in either type of catkin. A similar situation is observed for leaves, where *PtMPK7 *TA is more pronounced in mature leaves than in young leaves. This is similar to what has been reported for the rice putative ortholog *OsMAPK4 *(now annotated as *OsMPK7 *[39]), whose expression is higher in mature leaves than in young leaves [45].

#### Group D MPKs

The group D *MPKs *represent the largest group of *MPKs *in poplar, as they do in *Arabidopsis *and rice [4,39]. In rice and alfalfa (*Medicago sativa*), two group D *MAPK *genes (*OsBMWK1 *and *MsTDY1*) are induced transcriptionally by pathogen challenge and wounding, respectively [36,46] Activated OsBMWK1 (now annotated as OsMPK17-1 [39]) has also been shown to phosphorylate a transcription factor that binds a *cis*-acting element in the promoter of defence-related genes [47].

In poplar, *PtMPK17 *is the most highly expressed of the group D *MPK *genes and, indeed, it is the most highly expressed among all the *MPK *and *MKK *genes (Figure 9). On the other hand, the most weakly expressed group D *MPK *genes, *PtMPK9-1 *and *PtMPK9-2*, display low transcript levels in all organs with the noteworthy exception of all types of buds, and cell suspensions. As previously observed in other *MPK *groups, some members of the group D *MPK *genes seem to represent the paralogous products of recent genomic duplication events, since they possess a very high degree of sequence similarity, are located on different chromosomes and have only one direct putative ortholog in *Arabidopsis *[39]. *PtMPK16-1 *and *PtMPK16-2 *are particularly interesting paralogs, since they have very similar expression profiles in most organs, including male and female catkins. On the other hand, *PtMPK16-1 *is strongly expressed in male and female floral buds, whereas expression of *PtMPK16-2 *is barely detectable in these reproductive organs.

### MAPKKs

#### Group A MKKs

**Figure 10:**
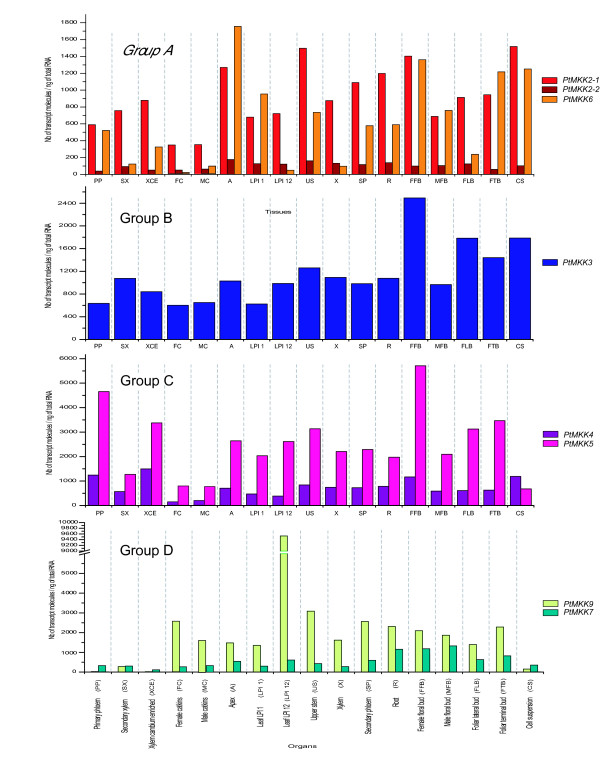
Steady-state transcript accumulation for all members of the four phylogenetic groups of *PtMKK *genes. After determination of RTqPCR primers efficiency and generation of standard curves, RTqPCR analysis was performed for each poplar *MAPKK *gene. Twelve nanograms of cDNA were used in each RTqPCR reaction. Results are expressed in number of specific transcripts per ng of total RNA. Values represent the mean of six independent reactions (two repeats for each of three independent samples). Cts were determined using single fluorescent readings that were taken after each cycle.

In other plant species, some group A *MKKs *appear to be functionally associated with group B *MPKs *[48,49] These phosphotransfer relationships have been involved in responses to abiotic stresses in *Arabidopsis*, and in cell development in tobacco. As in *Arabidopsis*, there are three group A *MKK *genes found in poplar. *PtMKK2-2 *shows low but constant levels of TA in all tested organs (Figure 10; see additional file 3), while the paralogous *PtMKK2-1 *is much more highly expressed. Higher expression of *PtMKK6*, on the other hand, seems to be associated with proliferating organs such as apex, floral and terminal buds, cell suspensions, and young leaves (LPI 1), with a 25-fold decrease in *PtMKK6 *expression levels observed along the foliar developmental gradient from young to mature leaves. Interestingly, the presumed *Arabidopsis *and tobacco orthologs of PtMKK6 have been involved in regulation of cytokinesis and cell division [13], suggesting that this protein may play an analogous role in poplar tissues.

#### Group B MKKs

Group B *MKKs *in poplar are represented by a single gene, *PtMKK3*. This is also seen in *Arabidopsis *(*AtMKK3*) and rice (*OsMKK3*) [39]. *MKKs *of this class are unique in encoding a characteristic MKK protein kinase domain fused in C-terminal to a putative nuclear transport factor 2 (NTF2) domain [4]. No biological functions have yet been assigned to plant MKK3s, but *PtMKK3 *has moderate to relatively high expression levels across all organs (TA between 400 to 2600), with the highest levels detected in female floral buds, lateral foliar buds and cell suspensions (Figure 10).

#### Group C MKKs

Among the plant MKKs, attention has been largely focused on those found in group C because of their demonstrated roles in stress signaling. Ectopic expression of constitutively-activated versions of the tobacco NtMEK2 protein, and of other group C MKKs, has been used to demonstrate that they are capable of phosphorylating and thus activating stress-responsive Group A MPKs [50-52]. Poplar possesses two group C *MKKs*, *PtMKK4 *and *PtMKK5*, both of which are expressed. Of the two, *PtMKK5 *shows higher TA in most organs, while the expression of *PtMKK4 *only predominates in suspension-cultured cells (Figure 10).

#### Group D MKKs

Limited functional information is available for group D *MKKs*, of which there are five encoded representatives in the poplar genome (*PtMKK7, PtMKK9, PtMKK10, PtMKK11-1 *and *PtMKK11-2*). However, our RTqPCR analysis suggests that only *PtMKK7 *and *PtMKK9 *are clearly expressed (Figure 10). The other three genes may therefore be pseudogenes, or be expressed only under circumstances that were not tested in our survey. The pseudogene hypothesis is also supported by the observation of structural differences in normally conserved motifs within the predicted PtMKK10, 11-1 and 11-2 protein kinase domains, and by the apparent absence of expression data for the *Arabidopsis *(*AtMKK10*) and rice (*OsMKK10-1/10-2*) putative orthologs [39].

While both *PtMKK7 *and *PtMKK9 *expression could be detected, the patterns differ significantly across organs and developmental stages (Figure 10). *PtMKK9 *expression is generally more pronounced except in secondary xylem, cell suspensions, primary phloem and xylem cambium-enriched, where *PtMKK7 *predominates. *PtMKK9 *is particularly highly expressed in mature leaves (LPI 12), where it reaches close to 10 000 transcript molecules per ng of total RNA, in contrast to the younger leaf sample (LPI1). The levels of transcript accumulation for *PtMKK7 *are on the other hand relatively constant across most organs. This striking difference in expression pattern has also been observed in *Arabidopsis *expression databases for *AtMKK7 *and *AtMKK9 *[53] where *AtMKK9 *transcription is most strongly associated with mature or senescing leaves.

## Discussion

The genomic organization of poplar *MAPK *and *MAPKK *genes clearly reflects the impact of whole genome duplications, of chromosomal duplications and of large-scale segmental duplications on the expansion of gene families. This is especially true for *Populus*, since genes represented by individual models in *Arabidopsis *are frequently found as unclustered paralogous gene sets in poplar. This mode of expansion is not unique to the *MAPK *and *MAPKK *genes, since a similar phenomenon has been observed for the poplar cellulose synthase (*CesA*) gene family [54]. The absence of tandemly duplicated gene clusters within the poplar *MAPK *and *MAPKK *gene families is also shared by other plant species including *Arabidopsis *and rice [39].

Comparative analysis of exon-intron junctions within the coding region of *Arabidopsis MAPK *and *MAPKK *gene families [55] and their poplar counterparts also highlights the conservation of these signaling components. Hence, group A and group C *MPKs *in both species display identical numbers of exons (six and two, respectively), and the sizes of the exons as well as the intron phases are extremely well conserved. These findings show that the respective degree of conservation of group A and group C *MPK *genes extends beyond primary sequence identity and is likely to be a feature of these genes in all eudicots.

A more complex situation exists for group B *MPKs*, where all group B *PtMPKs *contain six exons, while only two out of five group B *AtMPKs *(*AtMPK4 *and *AtMPK12*) display this organization. Nevertheless, except in *PtMPK5-1*, the phases of the various introns found in these gene models are also perfectly conserved. These gene models might thus share a common evolutionary history. On the other hand, *AtMPK5*, *AtMPK11 *and *AtMPK13 *all possess four exons with variable intron phase combinations. These combinations are not observed in any poplar *MPKs*. This suggests that the *Arabidopsis *group B *MPK *gene family has evolved differently than the corresponding poplar family. It is possible that one of the ancestral genes that gave rise to the present group B *AtMPK *family was not transmitted to poplar when these species diverged or that the precursor gene was lost during poplar evolution. Alternatively, the generation of the four exon configurations observed in *Arabidopsis *was the result of a duplication event that followed species divergence.

For group D *MPKs*, despite more complex configurations of exons and introns, it is possible to recognize that the respective putative orthologs between *Arabidopsis *and poplar display similar or sometimes even identical exon organization, with very well conserved exon length and intron phase. The only exception in this regard is *AtMPK15*, which possesses a unique seven exon composition within its coding region. This pattern is not observed for any other *Arabidopsis *or poplar *MPK *genes and indeed, no direct putative ortholog of *AtMPK15 *can be detected in the poplar genome. This *AtMPK *gene might therefore have arisen in *Arabidopsis *after species divergence, or might have been lost during poplar evolution.

At the level of *MAPKKs*, phylogenetic conservation of exon length and of exon-intron junctions is also generally observed. Hence, in group A *MKKs*, both the *AtMKK2 *and *AtMKK6 *coding regions are composed of eight exons, which is identical to the structure of their respective predicted orthologs in poplar, *PtMKK2-2 *and *PtMKK6*. In addition intron phase configuration is identical among these genes. On the other hand, the *AtMKK1 *coding region consists of six exons, a pattern that is not conserved in the coding region of the closest poplar putative ortholog, *PtMKK2-1*, which is constituted of nine exons. For group B *MKKs*, despite the high level of predicted protein sequence similarity between AtMKK3 and PtMKK3, the number of exons within their respective coding regions differs (eight exons for *AtMKK3*; nine for *PtMKK3*). However, this lack of conservation in the exon and intron organization is not caused by the kinase domain evolutionary status. In fact, the regions encoding the protein kinase domain are well conserved in terms of exon count, exon length and intron phase and differences between these two gene models are found at the end of the coding regions (within the NTF2 domain of the corresponding encoded proteins). Finally, as reported earlier [4], both the *Arabidopsis *group C and D *MKKs *display an intronless configuration. This trait is fully conserved within both poplar group C and group D *MKK *gene families.

Overall, exon lengths in both the *Arabidopsis *and poplar *MPKs *and *MKKs *are clearly more conserved than intron lengths. This indicates stronger negative selection for alteration of corresponding protein sequences. Additionally, most variations occurring in exon lengths come at the beginning or at the end of the coding sequences. This reflects that within the protein sequence, the centrally located kinase catalytic domain is probably submitted to more stringent functional conservation. Evolutionary analysis of plant terpene synthase genes also revealed strong conservation within the C-terminal enzyme catalytic site, with most variable domains observed in the N-terminal part of uncertain functions [56]. For their part, poplar *MPK *and *MKK *introns are generally much longer than those found in the corresponding *Arabidopsi*s genes. This may reflect reduced pressure for genome compaction within the larger poplar genome.

Transcript abundance at a given time and in particular organs is an important prerequisite to subsequent production of the corresponding protein required for proper execution of developmental, metabolic and signaling processes. The goal of the present study was to obtain a reasonably comprehensive overview of the whole organ expression patterns for all members of the poplar *MAPK *and *MAPKK *gene families. These families contain numerous recently duplicated members (paralogs), which raises the question of the extent to which these copies have remained functionally redundant. Our data suggest that while these gene families are highly conserved among eudicot species, individual family members are nevertheless evolving to display considerable diversity and specialization in the context of poplar biology. For example, while the paralogous genes *PtMPK3-1 *and *PtMPK3-2 *share 93% amino acid sequence similarity, and both genes are expressed at similar levels in most organs, *PtMPK3-2 *transcripts are markedly more abundant than those of its sister paralog in all four types of bud organ. This association with developmentally distinct juvenile organs suggests that *PtMPK3-2 *may be undergoing neo-functionalization for specific developmental roles, perhaps related to meristem development. For organs other than buds, the similar transcriptional activity of both *PtMPK3-1 *and *PtMPK3-2 *might simply represent genetic redundancy, where expression of both genes contributes to a common signaling pathway. Alternatively, this could be an example of sub-functionalization [57], where both genes have become compromised in some of their functions, but when operating together, can still provide the functionality associated with the ancestral gene. Since the closest *Arabidopsis *and tobacco presumed orthologs of *PtMPK3-1 *and *PtMPK3-2 *are *AtMPK3 *and *NtWIPK*, respectively, two stress-responsive MAPKs that respond at both the transcriptional and post-translational levels to biotic and abiotic stresses [58,15], it will be interesting to determine whether one or both poplar genes display similar defence-related functions.

The expression profiles of the paralogous *PtMPK6-1 *and *6-2 *genes differ from what has been reported for the putative orthologous tobacco genes, *NtSIPK *and *Ntf4*. While *NtSIPK *is expressed in leaves [19], stem and pollen [14], *Ntf4 *expression was found to be restricted to seeds, pollen and anthers [38] and notably, *Ntf4 *expression was not detected in female structures (ovaries and pistil) in the tobacco hermaphrodite flower. This suggests that specialized functions might have been acquired by one or both tobacco paralogs. On the other hand, since both paralogs are expressed in pollen, co-activation by the same MKK, NtMEK2, as well as the absence of phenotype after the silencing of *Ntf4 *[14], point to some level of genetic redundancy of these MAPKs in this particular organ. For poplar, despite the predominance in TA for *PtMPK6-1 *over *PtMPK6-2 *in all types of buds, in male and female flowers, there is no evidence of strikingly different patterns of expression. In fact, *PtMPK6-1 *and *PtMPK6-2 *display similar TA for most organs, suggestive of overall redundancy. Further investigation will be needed to evaluate the impact of this discrepancy in expression profiles between presumed orthologous tobacco and poplar genes. Interestingly, in a recent publication, NtSIPK and Ntf4 were both detected in leaf extracts using antibodies [59]. This contrasts with previously published data [38] and suggests that at least in leaves, both *NtSIPK *and *Ntf4 *display redundant expression profiles.

Expression of group B and C *MPK *genes has also been investigated in other species. For example, the phylogenetically closely related *Arabidopsis AtMPK1 *and *AtMPK2*, as well as the *Petunia hybrida PhMEK1 *gene and the tobacco *Ntf3 *gene, showed constitutive expression in various organs (leaves, stem, roots and flowers) [37,43,60]. Interestingly, *AtMPK2 *is more highly expressed than *AtMPK1 *in the analyzed organs [60], a situation also observed for the poplar predicted ortholog *PtMPK1 *over *PtMPK2*.

Other members of the group B and C *MPK *genes also display notable differences in their expression profiles. The poplar group C *PtMPK7 *is generally much more highly expressed than its paralog, *PtMPK14*, in all tested organs except cell cultures. As well, expression of the group B *PtMPK5-2 *is generally higher than that of *PtMPK5-1*. The physiological impact of these differences in levels of transcript accumulation among paralogs remains to be resolved. Such a pattern could however be an indicator of pseudogenization of one member of a recently duplicated gene pair, even though signal transduction genes are often thought to be subject to strong conservation constraints [61].

Although extensive microarray data are available, no systematic analysis of expression profiles of group D *MPK *genes in plants has been reported. However, *OsWJUMK1*, the rice putative ortholog of *PtMPK20-1/20-2*, was found to be expressed in both vegetative and reproductive organs [25] as are the poplar putative orthologs, with *PtMPK20-1 *expression dominating over *PtMPK20-2*. The *PtMPK16-1/16-2 *pair of paralogs is particularly interesting, since *PtMPK16-1 *is highly expressed in both male and female floral buds, whereas transcripts corresponding to *PtMPK16-2 *are barely detectable in these organs. Taken together with the *PtMPK3-1/3-2 *patterns, this points to particularly rapid divergence of functional specificity that highly similar paralogous genes appear to have evolved within the context of reproductive organ development in poplar.

Among the poplar *MKKs*, we found that *PtMKK6 *is strongly expressed in most of the actively proliferating organs we investigated. Consistent with this, the closest ortholog of *PtMKK6 *in tobacco, *NtMEK1*, encodes a protein that has been shown to be part of a MAPK cascade involved in the progression of the cell cycle [62]. A similar role has been identified for the *Arabidopsis *putative ortholog, AtMKK6 [13], which suggests that MKK6s could be highly conserved players in plant cytokinesis and stem cell development. *PtMKK3*, a group B *MKK*, is expressed at relatively high levels in all vegetative organs in poplar and is moderately expressed in all types of post-dormancy buds, as well as in male and female catkins. Likewise, in *Arabidopsis*, the orthologous *AtMKK3 *mRNA is detected in vegetative organs and also in the hermaphrodite flower [63].

## Conclusion

Overall, our work demonstrates that differential spatio-temporal transcript accumulation patterns exist for most members of both the *MPK *and the *MKK *gene families in poplar. Although virtually all poplar *MPK *and *MKK *genes are expressed during development, there are striking differences in steady-state TA in specific cases, some of which could be associated with functional divergence between recently duplicated paralogs. However, it is also critical to note that while RTqPCR data provide an estimate of whole organ gene expression levels, more pronounced local patterns of differential expression associated with regions composed of specialized cells will not be detected. More fine-grained analyses such as *in situ *hybridization or promoter:reporter phenotyping will be required to define the exact pattern of expression of these paralogous gene pairs, particularly in biological contexts uniquely relevant to dioecious woody perennials, such as gender specification, male and female flower maturation or wood formation. Finally, while the focus of the present study was on *MPKs *and *MKKs *expression profiling in a developmental context, plant MAPK cascade components also play central roles in responses to environmental cues. It will therefore be interesting to monitor the expression of these genes in physiological contexts such as tree defence signaling during biotrophic or necrotrophic pathogen infections, or adaptation to environmental stresses.

### Experimental procedures

#### Plant material and organ sampling

Hybrid poplar cell suspension cultures (*Populus trichocarpa *X *Populus deltoides *H11-11) were maintained as described previously [64]. Four days after transfer to fresh medium, cells were harvested by vacuum filtration, immediately frozen in liquid nitrogen and stored at -80°C to await analysis. Hybrid poplar cuttings (*P. trichocarpa *X *P. deltoides *H11-11) were grown in Promix soil under controlled greenhouse conditions (22°C/19°C day/night, 16-h day) for 2 weeks. Thereafter, young trees were placed in a growth chamber (Conviron, Environmental Growth Chambers Inc., Chagrin Falls, OH) where conditions were as follows: 26°C/22°C day/night, 20-h day, 60% RH, light intensity 100 μmol m^-2 ^s^-1^. Plants were fertilized once every 2 weeks alternatively with 10/52/10 (0.5 g/l) plus 15.5/0/0 (Ca 19% 0.5 g/l) or with 20/20/20 (1 g/l) until PI 16 stage was reached [65]. Apex, young (LPI 1) and mature (LPI 12) leaves, upper stem (10 cm below the apex), primary phloem and xylem (10 cm above the ground) and roots were simultaneously removed from the trees, frozen in liquid nitrogen and stored at -80°C. Each specific organ was harvested from 12 different trees. Three biological samples were thus created by pooling each organ sample from four different trees. Ten-year-old field-grown trees (*P. trichocarpa *X *P. deltoides*) located in Sainte-Croix-de-Lotbinière (46° 39'55"N 71° 51' 13" W) were used for sampling various bud types. Post-dormancy buds were collected on April 8 2005 on male and female trees by quickly removing them from previously cut branches. Bud samples were then flash frozen in liquid nitrogen and stored at -80°C. Other cut branches from male and female trees (*P. trichocarpa *X *P. deltoides*) were also brought back to greenhouses to induce bud flush using the following conditions; 22°C/19°C day/night, 16-h day. Branches were placed in plastic buckets half-filled with water and buds were allowed to develop. Male and female catkins (*P. trichocarpa *X *P. deltoides*) were collected when the completed developmental state was reached; 6 days for males and 11 days for females. At this stage, male catkins harbored pollenic bags and female catkins harbored fruiting capsules (Figure 7B,C). Other 10-year-old field-grown tree (*P. trichocarpa *X *P. deltoides*) organs (primary phloem, secondary xylem and xylem cambium-enriched) were kindly provided by Dr. Janice Cooke, Université Laval.

### RNA purification and amplification of 3' non-coding regions of poplar MPK and MKK genes

The poplar genomic sequences and predicted coding sequences are available from the DOE Joint Genome Institute database [66]. As described elsewhere [39] gene models for *MPKs *and *MKKs *were targeted for phylogenetic study and a nomenclature based on predicted protein sequence similarity with *Arabidopsis *and rice was adopted to reflect putative orthology. This information was also used to manually establish chromosome position, exon-intron junctions and intron phase for each *PtMPK *and *PtMKK *gene family member. Given the high degree of gene conservation among *MPK *and *MKK *genes, it was essential to develop gene-specific probes based on the respective 3' non-coding regions. To do this, a cDNA library was prepared from mRNA isolated from poplar cell suspensions. Frozen poplar cell suspensions were ground into powder in liquid nitrogen and RNA samples were prepared as previously described [67]. From 1.3 mg total RNA, mRNA was isolated and subsequently purified using the Oligotex mRNA Maxi kit (Qiagen, Mississauga ON). Subsequently, 3'-RACE PCR reactions were performed following the manufacturer's instructions (SMART™ RACE cDNA Amplification Kit, Clonetech, Palo Alto, CA). Based on the genomic and the predicted coding sequences of the poplar *MPK *and *MKK *genes, we designed one gene-specific sense primer at the end of the coding sequence of each gene (Tables 1 and 2). This allowed us to amplify the corresponding 3' untranslated region (UTR). Amplicons were analyzed by agarose gel electrophoresis, and these products were then ligated into pCR2.1 vector (Original TA Cloning Kit, Invitrogen, Carlsbad, CA), and electrotransformed into *E. coli XL1 blue *(Stratagene, La Jolla, CA). Plasmid DNA was subsequently purified using the QIA-prep-8 Plasmid Kit (Qiagen) and sequenced using the dideoxy nucleotide termination method with an ABI 373 Stretch XL sequencer (Applied Biosystems, Foster City, CA). 3'UTR sequences were aligned using Multalin [68] with the genomic and the predicted coding sequences of the corresponding poplar *MPK *and *MKK *genes.

All poplar organ-specific samples were ground into a powder in liquid nitrogen and total RNA was isolated as previously described [67]. To remove DNA traces in total RNA samples, we performed DNase treatments as recommended in the manufacturer's instructions (DNase 1 Deoxyribonuclease I digests, Invitrogen). cDNAs were synthesized from 500 ng total RNA (SuperScript™ III First-Strand Synthesis System, Invitrogen).

### Real-Time quantitative PCR (RTqPCR) analysis

In order to confirm the efficiencies of the RTqPCR primer sets, genomic DNA from *Populus trichocarpa *(Nisqually-1), *Populus trichocarpa X Populus deltoides *(H11-11) and *Populus deltoides *(ST-70) was extracted using 150 mg of leaf tissue (LPI 3). Briefly, plant material from each genetic background was ground into powder in liquid nitrogen. DNA extractions were then conducted using DNeasy system (Qiagen), according to the manufacturer's instructions. Five nanograms of DNA were used in each RTqPCR reaction that was carried out using the Opticon2 DNA Engine (MJResearch, Waltham, MA). After an initial 15 min activation step at 95°C, 45 cycles (94°C, 15 s; 57°C, 1 min; 72°C, 30 s) were performed and a single fluorescence reading was taken after each cycle immediately following the elongation period at 72°C. A melting curve was performed at the end of the cycling procedure to ensure single product amplification. Cycle threshold (Ct) values were determined by the Opticon Monitor 2 software at a manually set fluorescence threshold of 0.016. Agarose gel electrophoresis (1.2%) was also performed to compare amplicons obtained from the various genetic backgrounds.

To quantify organ-specific TA for the corresponding poplar *MPK *and *MKK *genes, standard curves were produced based on serial dilution of each of the pCR2.1 plasmids containing the cloned 3'UTR of each gene, and used to determine the number of transcript molecules as described in [69]. Twelve nanogram aliquots of cDNA were used in each RTqPCR reaction. Amplifications were conducted in 1× QuantiTect™ SYBR Green mix (Qiagen) with 0.25 μM of both forward (5') and reverse (3') oligonucleotide RTqPCR primers. Specific 5'- and 3'-oligonucleotides were designed to target the previously sequenced 3' UTR regions of each poplar *MPK *and *MKK *gene (Tables 1 and 2), and RTqPCR reactions were carried out as described above, except that the manually set fluorescence threshold was 0.03.

In order to obtain consistent and reproducible RTqPCR data, accurate measurement of RNA concentrations and the preparation of samples that are free from any inhibitors of the reverse transcription and PCR processes are critical steps. Moreover, since we have analyzed many different organs in parallel, it was necessary to normalize the data by employing internal standard candidate genes that show consistent expression over a wide array of tissues or organ samples. To validate proper dosage of cDNA, *cdc2 *RTqPCR primers were included in the analysis as a control gene. Primer sequences were as follows: forward primer sequence: 5'ATTCCCCAAGTGGCCTTCTAAG 3'; reverse primer sequence: 5' TATTCATGCTCCAAAGCACTCC 3'). *Act2 *was also employed as a control housekeeping gene and RTqPCR primer sequences were as follows: forward primer sequence: 5' TTCTACAAGTGCTTTGATGGTGAGTTC 3'; reverse primer sequence: 5' CTATTCGATACATAGAAGATCAGAATGTTC 3').

To simplify graphical presentation, standard deviations are not included in Figures 8 to 10. However, all the corresponding data together with respective standard deviation are available in the additional files 2 and 3. Each value of transcript accumulation for a specific *PtMPK *or *PtMKK *gene represents the average of six independent PCR reactions; two technical repetitions of three biological samples. Overall, regardless of organs or phylogenetic classification, technical variation within one sample was, on average, < 0.25 Ct. This means that our quantification of transcript accumulation was highly reproducible within one sample and that technical error has little impact on the values reported here. The variation from one biological sample to another within a specific organ was also consistently < 1.0 Ct. Samples that showed greater variation than 1.0 Ct were reamplified, with usually smaller disparity. The highest variations on transcripts numbers were found in leaf samples (LPI 1 and 12) as well as in xylem and secondary phloem, for both *MPK *and *MKK *gene families. The underlying reason for this finding remains unclear, but it could be associated with higher concentrations of potent inhibitors of the reverse transcription and/or PCR processes.

## Competing interests

The author(s) declare that they have no competing interests.

## Authors' contributions

M-CN and L-PH designed and performed all the *in silico *analysis and laboratory experiments, and drafted the manuscript. M-JM participated to the real time RT-qPCR experiments. NB and BEE critically revised the manuscript and provided important intellectual content. AS conceived the analysis, participated in its coordination and helped to draft the manuscript. All authors read, helped to edit, and approved the final manuscript.

## Supplementary Material

Additional File 1 Evaluation of each RTqPCR primer set efficiency using genomic DNA from three different genetic backgrounds: *Populus trichocarpa *(T), *Populus trichocarpa X Populus deltoides *(TXD) and *Populus deltoides *(D). Click here for file

Additional File 2 Raw values, standard deviations and means of the number of transcript molecules per ng of total RNA obtained for each *PtMPK *gene in the various poplar organs surveyed. Click here for file

Additional File 3 Raw values, standard deviations and means of the number of transcript molecules per ng of total RNA obtained for each *PtMKK *gene in the various poplar organs surveyed. Click here for file

## Figures and Tables

**Table 1:** Sequences of *PtMPKs *3'RACE and RTqPCR primers used for 3'UTR *MAPK *gene isolation and expression profiling.

**Group**	**Gene name**	**3' RACE Primers (5' to 3')**	**Forward RTqPCR Primers (5' to 3')**	**Reverse RTqPCR Primers (5' to 3')**
**A**	***PtMPK3-1***	CCCACTTGTTCACCCTCTGGCCATTG	CCATAAGCACTACCAGTCC	TCA GATACAGAGGAGTGC
	***PtMPK3-2***	AGCATTGGCTCATCCATACCTTGCAAGG	CTTTAGCACTACCAATCCCG	TGAACAATTAGCAAGTCCAGG
	***PtMPK6-1***	TCCCAACTGTCCACCCGGCAGCTATTG	CATTGTGACTGGTCAGCTTGA	GTTCCCCTGAATTCTGTCC
	***PtMPK6-2***	GATCCCAGACAGAGGATTACTGTTGAGG	ATGAGTCGACCAGCGTGGC	CACTACATTGACGCCGATAC
				
**B**	***PtMPK4***	AGTCGATGAGGCACTGTGCCATCCATAC	TGGTGTGCAGTTGGGAAGG	GAACCTTCATAGAGATTCTGC
	***PtMPK5-1***	TCGCATCACTGTTGATGAGGCCCTTTGT	CGTCATTTCCTGGAGAATATG	ACAATGCAATTCACTTCAGGC
	***PtMPK5-2***	CCCAATAACCGCATCACTGTCGATGAGG	CATAACTTCTGACACATTTGGG	CATACAACACATGATCCTTGC
	***PtMPK11***	GAGGCATTGTGCCATCCATACTTGGCAC	GTTTGCTGAGCAGAATCTCC	GCGTCCTCAAAACCATGAAG
				
**C**	***PtMPK1***	CCGGAGCACTGGAACACCCTTACATGTC	AAGAAGCACGCACAAGCCAG	TACAAGCTGAAAGGGCCACC
	***PtMPK2***	GCTGCAACCCTCCAGCTCAGGTCCC	GTGTGTTTTTGTACGTGTCTG	AGAACTAGAAGCTCACTGCC
	***PtMPK7***	CAGGGCTGTACGATCCAAGACGCGACCC	CTAGTGTGTCTGTGAGATCAG	GGATGCCATAGTCCGGCTC
	***PtMPK14***	ACCCTTACATGTCAGGGCTGCACGATCC	TGCACGATCCAAGACACGAC	TGCGAAAACAACTTCTGGGTG
				
**D**	***PtMPK9-1***	CCAGGGTCACAGCAACCAGATGGTTCAG	GATTCAGATGGCTGCCTTTG	ACAAGCTACTGCCTGATCTTC
	***PtMPK9-2***	GCAGCTCAAAGCACTGATATTGAGAGGC	CCAGCGTAGTGTGGATAGTG	TCACATCTCCTCTCTGCAATC
	***PtMPK16-1***	CGGTGCTGCGTTACAACAATTGTGGAGC	AATCTGTGCCATTTTGTCTGTG	TGGCCTTATGAATACAGCCAC
	***PtMPK16-2***	GTGGAGCAGCAGCAGCAGAGAATCTTGA	CAGCAGCAGAGAATCTTGAC	CCATTGGAACCTTCAACCAC
	***PtMPK17***	CTCTTTCTCGTGCAGCTAAGCAGAGCCC	CCTATTTGCACATCTTGGAGG	GCGGTAGTACATTTAGTGAGG
	***PtMPK18***	CCTCCCCAAACCAGCTCTCCACACCACTGCT	GATAAATCCTTTCCACCCTCG	GTTGACTACACCCTTGAAACC
	***PtMPK19***	CTGGGATGGCCATGGATGTGAACGCCTA	GTTGGTATCACGATCAATTATG	TGACTAGATACAGGACGTGG
	***PtMPK20-1***	GGGAGGGAGCAAGGAAGAACATGTGGAT	GGACTGAGGAGATCCACTTG	CCCCCTCTTTTTACACAATCC
	***PtMPK20-2***	GTGGCATGGCAGCCAAATATGCACCAGA	GGTTGGAGCTGTTCAATATGG	GACACGTTGATCTTCTGAGTC

**Table 2:** Sequences of *PtMKKs *" 3'RACE and RTqPCR primers used for 3'UTR *MAPKK *gene isolation and expression profiling.

**Group**	**Gene name**	**3' RACE Primers (5' to 3')**	**Forward RTqPCR Primers (5' to 3')**	**Reverse RTqPCR Primers (5' to 3')**
**A**	***PtMKK2-1***	TGGTATTGCTGGAGTGTGCAACAGGCCA	TGCAAATGCATCAAACTCCTG	CCACCCAAAAGAGACATCAG
	***PtMKK2-2***	CTCCACCTTCTGCACCACCAGACCAATT	AGCTTGCAAACTATGAGGAAC	CGATTCTGTCAGGGAAAACC
	***PtMKK6***	GCTGCAACCCTCCAGCTCAGGTCCC	GGGAAGGTTGTCATCTTTGG	TGGGTAATTCACAGGAGGTTC
				
**B**	***PtMKK3***	GCAGTTGCAATCCGCGTTTCAGGATCC	GCTCTATGTTCCTCCAGTTAC	GAAATGGTTTCATATCTGTCACG
				
**C**	***PtMKK4***	ATTGCTTGTTGTTTGCAGAGGGAGCCGG	GAATTGTTGTGGGTTATTGTTG	GGTCAGAACTAAAGGTGCTGTG
	***PtMKK5***	CCCAATAACCGCATCACTGTCGATGAGG	AGCATCCGTTTATTGTGAGAAG	AACTACCTACTAAGCAATTGGC
				
**D**	***PtMKK7***	CGAGCTTGCCGGAGGGAGCATCTGAGGA	TGACCCGAATGCCCAGTTAA	TTCCTCTCATTTCAAGAGCAG
	***PtMKK9***	GCGTCGGAGGAGTTTCGGGACTTTATTC	ACTTTATTCAGTGTTGCCTGC	GGGTCAAATCACAAGTCCAC
	***PtMKK10***	TGTGGAGAGAAACCAGACTGGGCAGCAT	TTTGGTCTGGATATAAGCTGTG	TGCAAAACCAGCATCATCTTC
	***PtMKK11-1***	GGGAGTGCTCTGCAGTTGTTGCAACACC	TGCAACACCCTTTTATACTGC	TGATGGAAGTGACAGGATGG
	***PtMKK11-2***	GGAGAATCCCCTAGCTTCCCAAAGGAAG	CCTATGTTTGCTTAGGGGAG	TTGGTCCAAGAGCCATTTCG
